# Short Stature on a Boy: Mosaicism with an Isodicentric Y Chromosome

**DOI:** 10.1155/2019/8563095

**Published:** 2019-04-14

**Authors:** Catarina Silvestre, Juliette Dupont, Rosário Silveira Santos, Brígida Robalo, Carla Pereira, Maria Lurdes Sampaio

**Affiliations:** ^1^Department of Endocrinology, Diabetes and Metabolism, Santa Maria Hospital, Lisbon, Portugal; ^2^Department of Human Genetics, Santa Maria Hospital, Lisbon, Portugal; ^3^Pediatric Endocrinology Unit from Department of Pediatrics, Santa Maria Hospital, Lisbon, Portugal

## Abstract

Mosaicism brings great variability into the clinical expression of numerical and structural chromosomal abnormalities. The phenotypic variability of 45,X/46,XY mosaicism extends from Turner syndrome to apparently physically normal males. We present a case of a 14-year-old adolescent with short stature and delayed puberty, who was admitted in a Paediatric Endocrinology outpatient clinic. After a careful investigation, he was found to have a 45,X/46,X,idic(Y)(p11.32) mosaicism. This case report emphasizes the wide range of etiologies that can be involved in short stature and that chromosomal study is an important tool when firstly approaching males with short stature, avoiding unnecessary tests. There is an important clinical need for gonadal follow-up in this situation and for support in the decision about sex of rearing and sex orientation, when justifiable.

## 1. Background

Y chromosome is important for male development as it contains key genes such as sex-determining gene *SRY* (sex-determining region Y) and spermatogenesis genes. Structural abnormalities of the Y chromosome include rings, deletions, inversions, and dicentric chromosomes [1]. The latter are the most commonly reported structural abnormality and can be caused by break in the short arm, thus having two copies of the Y long arm, idic(Y)(p), or by break in the long arm, thus having two copies of the short arm, idic(Y)(q), which may or may not contain *SRY* gene [2–4].

Isodicentric chromosomes originate during spermatogenesis by aberrant homologous crossing-over between opposite arms [5]. These isodicentric chromosomes contain two centromeres, resulting in mitotic instability and leading to loss of the Y chromosome. The clinical consequence is the presence of an additional cell line, normally 45,X [1–3, 5, 6]. The majority of patients with isodicentric Y chromosomes exhibit short stature and various disorders of sexual development. The phenotype is heterogeneous, ranging from women with Turner syndrome to patients with sexual ambiguity to men with only spermatogenesis abnormalities [1–3, 5–7]. A comprehensive review of the literature allows to state that sexual development variability in these patients depend on two factors: dicentric Y chromosome structure and mosaicism distribution on the various organs and systems [2].

We present a clinical case of a young male with short stature and delayed puberty, with 45,X/46,X,idic(Y)(p11.32) mosaicism, described according to the ISCN 2016 standard (International System for Human Cytogenetic Nomenclature [4]).

## 2. Case Report

A 14-year-old boy was referred to our Paediatric Endocrinology Center due to short stature. He was the first son of two children, with a healthy sister and irrelevant familial history. Familial target height was on percentile 3–10. Uneventful pregnancy, delivery, and neonatal period were seen. At the age of 5, he was submitted to correction of aortic coarctation and had arterial hypertension diagnosis, beginning treatment with enalapril (follow-up at Cardiology). He had an adequate psychomotor development. Height growth was on percentile 10–25 until he was 11, with growth deceleration since then. On first appointment with pediatric endocrinology, the patient's height and growth velocity were below percentile 3. His weight evolution was on percentile 25–50 until the age of 7, with exponential rise afterwards until percentile 97 (body mass index of 31 kg/m^2^).

At observation, besides height and weight disproportion already mentioned (weight 62 kg and height 141.2 cm), the patient presented round and red face, large and short neck, cervical *acanthosis nigricans*, well-muscled body, and melanocytic nevus on the back and limbs. Pubertal development: axillary hair present; pubic hair at Tanner stage 2; penis covered by prepubic fat (length 5.5 cm) but normal consistency; and testis in the scrotum, with a bilateral testicular volume of 4 ml^3^.

Laboratorial and imagiologic evaluation:

- Blood count, albumin, renal and hepatic function, ionogram, and phosphocalcic metabolism were normal
- Lipidic and glucidic profile: total cholesterol 219 mg/dL (reference range: <200 mg/dL), HDL 55 mg/dL (r.r.: >60 mg/dL), LDL 141 mg/dL (r.r.: <130 mg/dL); triglycerides 115 mg/dL (r.r.: <150 mg/dL); HbA1c 5.8%; glucose/insulin ratio 3.7 (low, suggestive of insulin resistance)
- Celiac disease: negative anti-gliadin and anti-transglutaminase antibody measurements
- Thyroid function was normal
- Adrenal function evaluation: normal basal 17-hydroxyprogesterone and dehydroepiandrosterone-sulfate for Tanner stage 2. Normal basal and stimulated values for 17-hydroxyprogesterone, 11-deoxycortisol, and delta-4-androstenedione in ACTH stimulation test
- IGF-1 and IGFBP3 were normal
- Left hand and wrist X-ray: 15-year-old bone age, for a chronologic age of 14 years and 4 months
- Renal ultrasound: no anomalies

Considering growth deceleration, arterial hypertension, round and red face, *acanthosis nigricans*, and hypercholesterolemia, the first hypothesis was hypercortisolism. However, 24 h urinary cortisol was normal (230 *μ*g/24 h, to r.r.: 55.5–286 *μ*g/24 h) as well as overnight 1 mg-dexamethasone suppression test (0.6 mg/dL, to r.r.:<1.8 mg/dL).

Because there was growth deceleration and nonevolving puberty, as well as an advance in the bone age, pituitary-gonadal axis was evaluated: normal prolactin measurement, undetectable gonadotrophins (LH and FSH), and total testosterone determinations, which could be compatible with a prepubertal stage or hypogonadotropic hypogonadism. Cranial magnetic resonance imaging showed no anomalies. In order to differentiate hypothalamic or pituitary origin for this hypogonadism, the next step should have been the LH-RH test. However, this was not performed as the patient started exogenous testosterone after seeking a second medical opinion.

**Figure 1:**
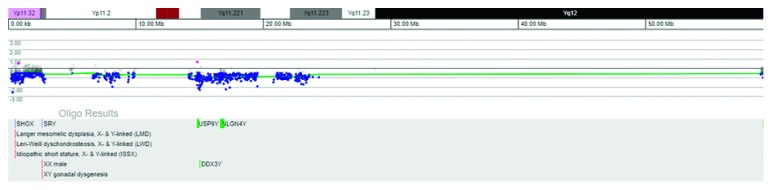
ArrayCGH analysis of the patient peripheral blood DNA hybridised against a normal male control shows a total deletion of the Y chromosome (58.80 Mb), but not of the X chromosome (data not shown).

**Figure 2:**
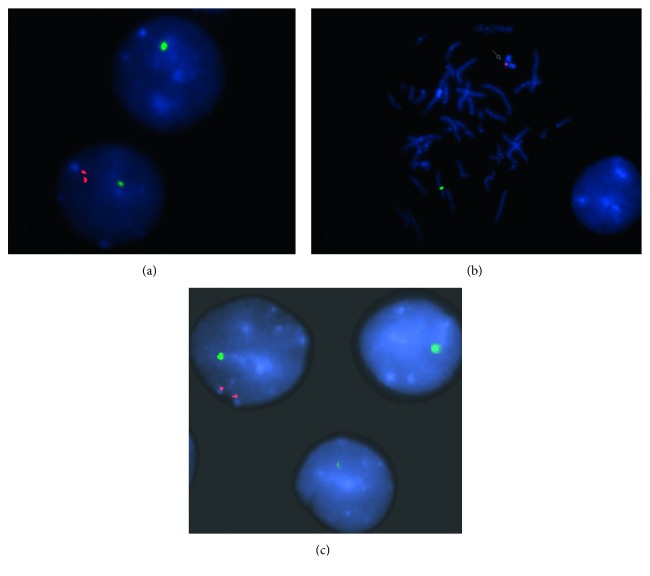
(a) FISH analysis depicting two interphase cells. Top: a cell showing only one green signal for the centromeric X region (DXZ1) indicating a 45,X cell; bottom: the cell shows one green signal (DXZ1) and two distinct but proximal red signals for the Y centromeric region (DYZ3) present on the idic (Y) chromosome. (b) FISH analysis using probes for the centromeric X region (DXZ1, green) and for the sex-determining region of Y chromosome on Yp11.3 (SRY, red) on a metaphase spread showing one signal on the chromosome X and one condensed signal on the idic (Y) (arrow) and (c) on interphase cells showing two hybridizations signals for the SRY region in mosaic.

**Figure 3:**
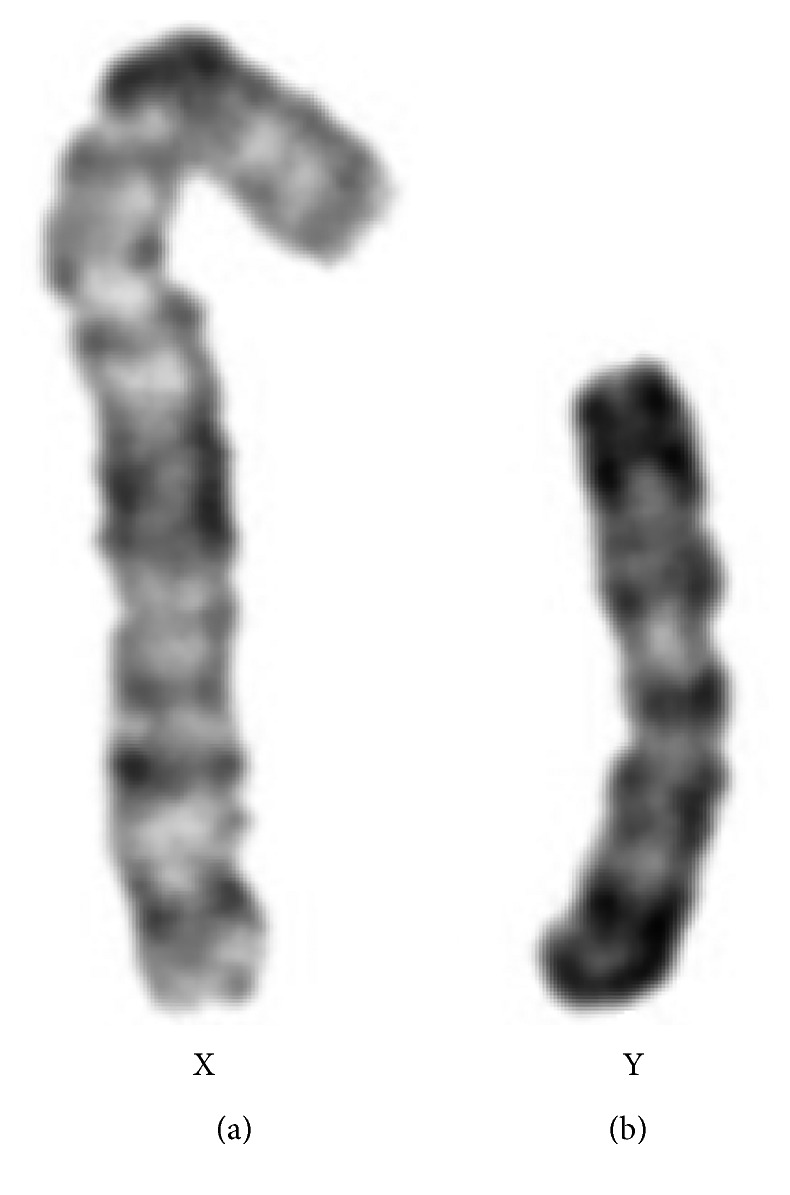
Patient's partial karyotype (GTL banding) showing the normal chromosome X (a) and the idic(Y)(p11.3) (b).

The patient was then referred for medical genetic evaluation, and a molecular analysis was requested. ArrayCGH (Comparative genomic hybridization, PerkinElmer® CGX-HD 180K, Genoglyphix v3.1) identified a mosaicism involving chromosome Y (Figure 1). This rearrangement was further characterized by karyotype and FISH (fluorescence in situ hybridization) with probes for the SRY and for the X (DXZ1) and Y (DYZ3) centromeric regions (Cytocell©) in the blood and buccal mucosa (Figures 2 and 3). This procedure confirmed the existence of two cell lines:

- a major line with a single hybridization signal for the chromosome X centromeric region, i.e., with 45 chromosomes and no Y chromosome, present in 72% and 51% of the lymphocytes and oral epithelial cells, respectively.
- a minor line with a hybridization signal for chromosome X centromeric region and a double hybridization signal for Y centromeric region compatible with a dicentric chromosome, present in 28% and 49% of the cells of peripheral blood and the oral mucosa, respectively. To further characterize this rearrangement, an SRY probe was used and a double hybridization signal for the Yp11.3 region was detected, at interphase. At metaphase, this minority line shows only a condensed hybridization signal for the SRY locus on the isodicentric chromosome, confirming the absence of only a small part of the short-arm terminal region distal to Yp11.32. In other words, this line is formed by 46 chromosomes with a structurally modified Y constituted by two long arms and part of the small arm, with loss of short arm terminal region at Yp11.3-idic(Y)(p11.3).

Together with the arrayCGH findings, the patient karyotype was

- mos 46,X,idic(Y)(p11.3)[12]/45,X[10].ish idic(Y)(SRY+).nuc ish (DXZ1x1)[300/415]/(DXZ1x1,SRYx2)[115/415].arr[GRCh37] Xp22.33/Yp11.32(296520_1211406)x0∼1,Yp11.32q12(246520_59049419)x0∼1

Considering this mosaicism, a testicular ultrasound (US) was performed with no anomalies detected.

Nowadays, the patient is kept under surveillance in Pediatric Endocrinology, under therapeutics with 200 mg testosterone enanthate (intramuscular) monthly. A secondary sexual characteristics progression was observed: development of axillary and pubic hair and testicular volume growth to 8 ml^3^. On last appointment, the patient was 68.7 kg and 144.5 cm tall. There has been nutritional and regular physical activity counselling as well as natural vegetable steroid ingestion encouragement in order to control obesity and dyslipidemia.

In the future, it is crucial to maintain follow-up and early detection of potential gonadic alterations, with regular testicular US (there is no consensus on periodicity, but the majority recommends annual evaluation; when in doubt a testicular biopsy should be performed), and preconception genetic counselling.

## 3. Discussion

The term “disorders of sexual development” (DSD) refers to congenital conditions in which development of chromosomal, gonadal, and/or anatomical sex is atypical. Nowadays, this set of pathologies can be subdivided into three main groups in order to simplify clinical evaluation [8, 9]:

1. Sex chromosome DSD
2. 46,XY DSD
3. 46,XX DSD

The first group englobes a condition known as mixed gonadal dysgenesis characterized most of the time by asymmetric testicular development; commonly unilateral testis and contralateral streak gonad are observed. Also, müllerian structures persistence and different degrees of masculinization can be observed. Although it is associated to various karyotypes, 45,X/46,XY mosaicism is most frequent, found in 35% of these patients [10, 11].

Mosaicism induces a highly variable phenotype; 45,X/46,XY mosaicism can be observed in Turner syndrome patients, mixed gonadal dysgenesis and, furthermore, apparently normal men just like the reported case. Clinical manifestations can range from partial virilisation and genital ambiguity at birth to patients with complete female or male phenotype. Sexual determination in these patients with mosaicism is dependent on the dominant cell line in undifferentiated gonads, i.e., 45,X presence gives rise to Turner syndrome, 46,XY presence gives rise to male phenotype, and existence of both lines originate mixed gonadal dysgenesis.

The presence of cell lines with 45,X is frequently associated with rearranged Y chromosomes which, in turn, also influence phenotype [5, 11, 12]. It is known from studies with transgenic mice that the presence of *SRY* gene alone is sufficient to initiate testicular development. Thus, the presence or absence of *SRY* gene in an abnormal Y chromosome constitutes another factor of phenotypic diversity [5]. The structural anomaly of Y chromosome, isodicentric, detected in the present case has a particularity despite being one of the most common anomalies of Y chromosome. The level at which the breakpoint on the short arm was identified, Yp11.32, is unusual, with a few cases published: four with Turner syndrome phenotype (karyotype 45,X/46,X,idic (Y)) [2, 13–15], three azoospermic men (two with 45,X/46,X,idic(Y) karyotype [16, 17] and other with 46,XY/46,X,idic(Y) karyotype [18]), and one with mixed gonadal dysgenesis [19] and hypospadia [20]. Structure of dicentric Y isochromosome, Yp and Yq breakpoints and the level of mosaicism and distribution constitute variables responsible for phenotype definition. Nonetheless, difficulty in establishing an adequate phenotype-karyotype correlation remains [18].

Mosaic 45,X/46,XY individual's stature may be normal or short. The cause of growth defect is not clearly defined; however, *homeobox* short stature absence–containing *SHOX* gene–is considered the most probable cause. Mosaicism 45,X/46,XY male patients usually respond to growth hormone therapy, in a similar way as female individuals, as long as precocious diagnosis is done [21]. In this particular case, short stature is explained by *SHOX* gene haploinsufficiency in the pseudoautosomal region of the Y chromosome's short arm. Therefore, this case highlights the importance of karyotype evaluation when studying short stature etiology in individuals with male phenotype.

**Figure 4:**
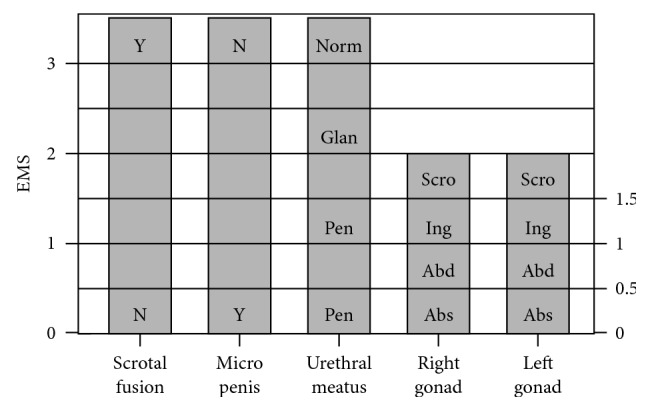
External masculinization score (EMS) based on the position of the gonads, length of the phallus, presence of scrotal fusion, and position of the urethral meatus. Y: yes; N: no; Norm: normal; Glan: glanular; Pen: penile shaft; Pe: perineal; Scro: scrotal sac; Ing: inguinal; Abd: abdominal; Abs: absent (adapted from Brook's Clinical Pediatric Endocrinology).

During the follow-up period, there are three main problems: risk of gonadoblastoma/dysgerminoma development, gender assignment/sex of rearing, and fertility. Concerning the first issue, it is known that Y chromosome material seems to take part in gonadoblastoma tumorigenesis. In 45,X/46,XY mosaicism patients belonging to female gender or with sexual ambiguity, the risk of developing gonadoblastoma is approximately 15–27%. This risk may be lower when streak gonads or near-normal testicular structure are present. Thus, preventive laparoscopy and gonadectomy are recommended in female patients that have Y chromosome material. In the case of male patients, gonadectomy may be considered when there is sexual ambiguity, like hypospadias or cryptorchidism [2, 12, 20, 21]. The presence of *SRY* gene seems to have a protective role against malignization because of the near-normal testicular development [5]. Application of *external masculinization score* (*EMS*; higher EMS scores correlate with a more advanced male genital differentiation (Figure 4)) may guide and define gonadal differentiation. Also, it helps at malignancy risk evaluation in 45,X/46,XY mosaicism patients: there is an inverse relationship between EMS score and gonadal malignization risk [22]. Current recommendations support testicular biopsy on gonadal dysgenesis cases, at puberty, seeking signs of premalignant lesions, i.e., *carcinoma in situ* [6].

In terms of sex rearing, it may constitute the most difficult obstacle to manage, psychologically and emotionally speaking, mainly in a patient going through puberty. There are various factors involved in this process; nevertheless, both external genitalia virilisation degree and the presence of gonads with testicular characteristics are the ones with the greatest impact [21]. According to some publications, for EMS with higher scores, patients identify themselves as belonging to male gender, while patients who recognize themselves as being female have lower scores [21]. Psychological counselling should be provided in these situations when necessary. In agreement with current concepts and consensus statements, preconception genetic counselling and reproductive medicine examination at adulthood are recommended.

In the present case, since external genitals are masculine and the patient was sure about his gender assignment, this kind of support was not necessary.

Lastly, in terms of future fertility, it is difficult to establish an accurate phenotype-karyotype correlation. The wide forms of phenotype presentations published so far show us the unpredicted fertility potential [12–18]. As stated before by Guttenbach et al. and Codina-Pascual et al., the existence of azoospermic males with a 45,X cell line and only a few 46,XY cells does not support the idea of a clear association between female phenotype and the proportion of 45,X cell lines. Instead, it depends on frequency and tissue distribution of cell lines present in mosaic form [18]. However, the fact that our patient had initially a low testicular volume may predict a deficiency of *Sertoli* cells. At adult age, it is beneficial to perform a spermogram after switching exogenous testosterone for gonadotrophic therapy, aiming for a near-optimal result.

With this case, the authors intend to illustrate the difficulty throughout short stature's investigation in Pediatrics. It also highlights karyotype study relevance which should be requested not only to female patients suspected of having Turner syndrome but also to male patients mainly with external genitalia or puberty alterations. Lastly, it is important to emphasize gonadal malignancy risk surveillance and sex rearing in these patients.

## Additional Points

*Learning Points/What Is New*. Karyotype studying may be extremely important when evaluating short stature in male patients. 45,X/46,XY mosaicism presence leads to a variable disturbance of sexual development, from women with Turner syndrome to sexual ambiguity, as well as men with spermatogenesis anomaly. Gonadal surveillance should be regular in order to early detect gonadoblastoma development.Besides the challenge of diagnosis and management of the disease, it is also important to highlight the need to support sex rearing and gender identity in these patients
